# Identification and optimisation of next generation inhibitors of IDO1 and TDO

**DOI:** 10.1186/2051-1426-3-S2-P422

**Published:** 2015-11-04

**Authors:** Alan Wise, Phillip Cowley, Barry McGuinness, Sarah E Trewick, Thomas Brown

**Affiliations:** 1 IOmet Pharma Ltd, Edinburgh, UK

## Introduction

The enzymes tryptophan 2,3-dioxygenase (TDO) and indoleamine 2,3-dioxygenase (IDO1) catalyse oxidation of the essential amino acid tryptophan (Trp) leading to the formation of immunosuppressive kynurenine (Kyn) pathway metabolites that dampen the immune response in the tumour microenvironment. Both IDO1 and TDO have been shown to be up-regulated in a variety of cancers and blockade of their activity has been shown to stimulate the anti-tumour immune response in pre-clinical animal models. We have discovered multiple novel chemical series of both highly selective and dual-acting inhibitors of IDO1 and TDO. Herein we describe their *in vitro* and *in vivo* characterisation.

## Methods

*In vitro* assays measured the effects of TDO/IDO1 inhibitors on Kyn production in cancer cells, hPBMCs and effects on T cell proliferation in co-culture systems. *In vitro* ADME properties and *in vivo* PK/PD profiles were measured by standard methods. In the LPS model of Kyn stimulation, LPS was administered at 5 mg/kg IP simultaneously with oral administration of IDO1 inhibitors. Plasma and tissue samples were collected 20 hours after dosing.

## Results

We have identified multiple distinct chemical series of novel IDO1 and TDO inhibitors which demonstrate nM potencies. In addition, we have also identified dual-acting TDO and IDO1 inhibitors, exemplars of which exhibit nM potency at each enzyme. Such dual-acting molecules are required to fully inhibit Kyn levels in IFNγ-stimulated A172 glioblastoma cells expressing both enzymes, demonstrating the utility of dual enzyme blockade in cancer cells. The compounds also relieve inhibition of T cell proliferation in cancer cell/T cell co-culture assays and demonstrate highly favourable physico-chemical and pharmacokinetic properties. These properties translate to superior PK/PD effects following oral dosing in rodents with profound and sustained modulation of plasma Kyn and Trp levels. In a mouse model of LPS-induced IDO1 activation, our highly selective IDO1 inhibitors fully ablate the elevation of Kyn in both plasma and lung tissues in a dose-dependent manner. This lung Kyn modulation is more potent and efficacious than that observed for molecules in clinical trials.

## Conclusions

We describe the characterisation of multiple novel chemical series of potent, selective and dual-acting TDO and IDO1 inhibitors. The drug-like properties of these potent molecules translate to superior *in vivo* PK/PD profiles compared to compounds in clinical trials. This clear metabolic advantage is expected to translate to a significantly better PK/PD effect in humans, especially in lung cancer – a major opportunity for a-PD-1/PD-L1 and IDO1 inhibitor combination therapy.

